# Direct Observation of Dimerization between Different CREB1 Isoforms in a Living Cell

**DOI:** 10.1371/journal.pone.0020285

**Published:** 2011-06-01

**Authors:** Hisayo Sadamoto, Kenta Saito, Hideki Muto, Masataka Kinjo, Etsuro Ito

**Affiliations:** 1 Laboratory of Functional Biology, Kagawa School of Pharmaceutical Sciences, Tokushima Bunri University, Sanuki, Japan; 2 Nikon Imaging Center, Research Institute for Electronic Science, Hokkaido University, Sapporo, Japan; 3 Laboratory of Molecular Cell Dynamics, Faculty of Advanced Life Science, Hokkaido University, Sapporo, Japan; National Institute of Health, United States of America

## Abstract

Cyclic AMP-responsive element binding protein 1 (CREB1) plays multiple functions as a transcription factor in gene regulation. CREB1 proteins are also known to be expressed in several spliced isoforms that act as transcriptional activators or repressors. The activator isoforms, possessing the functional domains for kinase induction and for interaction with other transcriptional regulators, act as transcriptional activators. On the other hand, some isoforms, lacking those functional domains, are reported to be repressors that make heterodimers with activator isoforms. The complex and ingenious function for CREB1 arises in part from the variation in their spliced isoforms, which allows them to interact with each other. To date, however, the dimerization between the activator and repressor isoforms has not yet been proved directly in living cells. In this study, we applied fluorescence cross-correlation spectroscopy (FCCS) to demonstrate direct observation of dimerization between CREB1 activator and repressor. The FCCS is a well established spectroscopic method to determine the interaction between the different fluorescent molecules in the aqueous condition. Using differently labeled CREB1 isoforms, we successfully observed the interaction of CREB1 activator and repressor via dimerization in the nuclei of cultured cells. As a result, we confirmed the formation of heterodimer between CREB1 activator and repressor isoforms in living cells.

## Introduction

Cyclic AMP-responsive element binding protein (CREB) family protein is a transcription factor that plays a critical role in regulating numerous biological systems, such as development, cancer, and memory formation [1], [2]. One of the CREB family proteins CREB1 can activate gene expression in a phosphorylation-dependent manner, and CREB1 binds to a cyclic AMP-responsive element (CRE) in the promoter regions of target genes as a dimer [3]. CREB1 contains three functional regions: (1) a phosphorylation site (P-box or kinase-inducible domain, KID), (2) a glutamine-rich transactivation domain (Q-domain), and (3) a leucine zipper (bZIP) domain. The P-box is the target region affected by several different kinase cascades and is required for the induction of gene expression; the Q-domain interacts with a component of the TFIID complex for basal transcription; and the bZIP domain is necessary for DNA binding and dimerization. Another remarkable feature of CREB1 is the variability of spliced isoforms, giving rise to functionally different CREB1 proteins with either activating or repressing potential on target gene expression [4]–[6]. The activator isoform contains all three functional regions. On the other hand, the repressor isoform does not include either the P-box or the Q-domain but does possess the bZIP domain. Because the repressor maintains the bZIP domain, they have been expected to interact with the activator and to interfere with gene induction via heterodimerization. Previous reports have revealed the existence of CREB1 spliced isoforms and the gene regulation by the splicing products in various animal species [5], [7]–[10].

To date, however, there has been no direct observation to determine whether a heterodimer can be formed between the CREB1 activator and the repressor in living cells. In our recent studies, we comprehensively analyzed an alternative splicing of the CREB1 gene in the mollusk *Lymnaea stagnalis* [11]. *Lymnaea* CREB1 gene in the brain was expressed as seven spliced isoforms encoding two protein isoforms, either the CREB1 activator or repressor. Using the two spliced products, we here examined the dimerization between different CREB1 isoforms to assess the quantitative analysis of protein interaction. To determine whether or not different CREB1 spliced isoforms interact with each other, we applied fluorescence cross-correlation spectroscopy (FCCS) analysis, a confocal microscope-based method. FCCS is a well-investigated method for observing direct associations between differently labeled proteins in femto-liter confocal volumes, and it enables us to directly and noninvasively observe the interaction between proteins in a living cell [12]–[14]. As a result, we first confirmed the strong interaction between CREB1 activator and repressor isoform proteins via the bZIP domains in the nuclei of living cells. Our present findings suggest that the CREB1 spliced isoforms, transcriptional activator, and repressor interact with each other continuously, resulting in the complexity and diversity of CREB-mediated gene regulation via the formation of their homodimer and heterodimer in the nuclei of living cells.

## Results

### Expression of CREB1 isoform proteins in living cells

To examine microscopically the interaction between CREB1 isoforms, we here used two different CREB1 isoform proteins of the pond snail *Lymnaea stagnalis*. The *Lymnaea* CREB1 isoforms were applied because we had already finished a comprehensive analysis of alternative splicing [11]. Seven splicing variants of CREB1 mRNA were identified and their sequence characterization allowed them to be grouped into only two protein isoforms: (1) the CREB1 activator (264 amino acids) containing both a phosphorylation site (P-box) and a leucine zipper (bZIP) domain; and (2) the CREB1 repressor (167 amino acids), containing a bZIP domain but not a P-box, that might be able to make a heterodimer with the activator protein. And heterodimerization will result in interference with gene induction because of the lack of a P-box.

**Figure 1:**
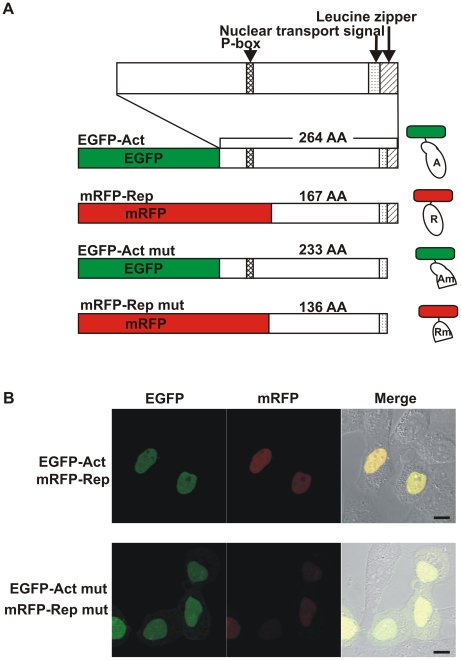
Expression of fluorescent-labeled CREB1 isoform proteins in HeLa cells. A, Expression constructs. Each CREB1 activator (Act) or truncated activator (Act mut) proteins was fused with EGFP (green box), and each CREB1 repressor (Rep) or truncated repressor (Rep mut) protein was fused to tandem mRFP (red box). Within CREB1 isoform proteins, the functional domains are shown as patterned boxes. Schematic diagram of the fluorescent-labeled CREB1 isoform proteins are shown on the right side. B, Confocal microscopy images of HeLa cells transfected with expression constructs encoding EGFP- (green) or mRFP- (red) labeled CREB1 isoform proteins. CREB1 isoform proteins (EGFP-Act and mRFP-Rep, top) or truncated CREB1 proteins (EGFP-Act mut and mRFP-Rep mut, bottom) were cotransfected, and the cells were imaged 16 h after transfection. Scale bars, 10 µm.

We constructed four types of plasmids with two different fluorescent proteins for labeling: enhanced green fluorescent protein (EGFP) and tandem monomeric red fluorescent protein (mRFP) (Fig. 1A). Following a previous report [12], we used tandem mRFP to improve the weak brightness of mRFP. The CREB1 activator protein was fused to EGFP (EGFP-Act), whereas the CREB1 repressor protein was fused to mRFP (mRFP-Rep). Because the dimerization domains (bZIP domains) of the CREB1 isoform proteins exist at the C-terminal part, the expression constructs code for the N-terminal fusions of CREB1 activator or repressor isoform proteins with EGFP or tandem mRFP, respectively, to protect the dimerization ability of CREB1 isoform proteins. For negative control experiments, we also constructed plasmids encoding truncated CREB1 proteins (EGFP-Act mut and mRFP-Rep mut). The dimerization domains of these proteins were selectively truncated without affecting the nucleus translocation signal (Fig. 1A).

We then transfected the expression constructs into the human tumor cell line HeLa. Sixteen hours after transfection, transiently expressed CREB1 isoform proteins, EGFP-Act and mRFP-Rep, were observed in the nuclei of the cells, which we expected because of their nuclear transport signals (Fig. 1B, EGFP-Act, mRFP-Rep). Some cells showed deformation of the nuclei, but no cell damage, such as cell death or a significant number of changes, was observed. This is quite similar to the case with the control cells transfected with the EGFP-C1 vector (data not shown). Thus, the biological activities of HeLa cells did not seem to be affected by the expression of *Lymnaea* CREB1 isoform proteins. In the case of the plasmids of EGFP-Act mut and mRFP-Rep mut, fluorescence microscopy revealed that these truncated CREB1 proteins were also localized mainly in the nucleus, but partly in the cytoplasm (Fig. 1B, EGFP-Act mut, mRFP-Rep mut).

### FCCS analysis of direct interaction between CREB1 isoform proteins in living cells

**Figure 2:**
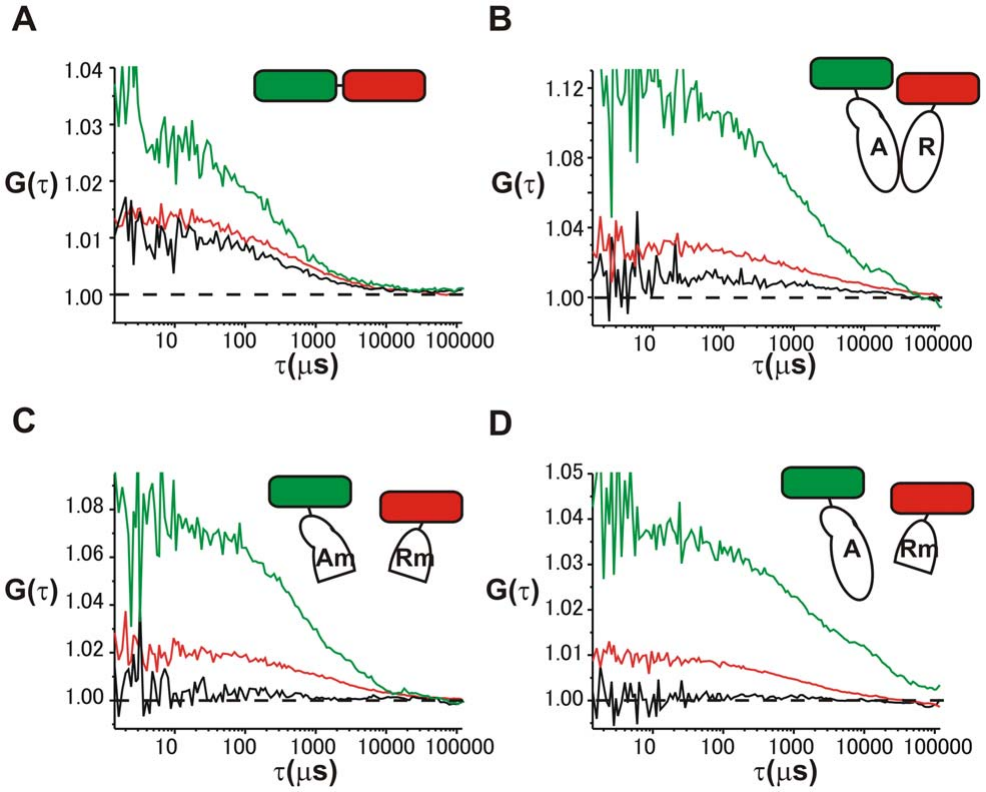
FCCS measurement of differently labeled CREB1 isoforms in the nuclei of living cells. The inset is a schematic diagram showing fluorescent-labeled proteins used with each experiment. Auto- and cross-correlation curves of fluorescent-labeled proteins were shown for EGFP- mRFP chimera protein (A), EGFP-Act and mRFP-Rep (B), EGFP-Act mut and mRFP-Rep mut(C), and EGFP-Act and mRFP-Rep mut (D), respectively. The green curve denotes the auto-correlation of the green channel [*G*g(τ)], the red curve the auto-correlation in the red channel [*G*r(τ)] and the black curve the cross-correlation curve [*G*c(τ)].

To investigate the molecular interactions between CREB1 activator and repressor isoform proteins, we applied dual-color fluorescence cross-correlation spectroscopy (FCCS) to living cells. Typical auto-correlation curves and cross-correlation curve of FCCS data were shown in Figure 2. As a positive control, we measured the cross-correlation for the interaction between EGFP and mRFP fluorescent molecules using EGFP-mRFP fusion proteins (EGFP-mRFP chimera, Fig. 2A). The interaction between different CREB1 isoforms was also examined using fluorescent-labeled CREB1 activator and repressor proteins (EGFP-Act and mRFP-Rep, Fig. 2B). For one of the negative control experiments, we applied CREB1 isoforms lacking the dimerization domains (EGFP-Act mut and mRFP-Rep mut, Fig. 2C). Further, to ensure that the dimerization domain was not responsible for nonspecific interaction between CREB1 isoforms, we also performed another negative control experiment using EGFP-labeled CREB1 activator (EGFP-Act) that possesses the ability of dimerization (EGFP-Act and mRFP-Rep mut, Fig. 2D). For each measurement, we concerned to keep the ratio of expressed proteins constant to accurately examine the interaction between EGFP-labeled and mRFP-labeled proteins. For the positive control, EGFP and tandem mRFP fusion protein (EGFP-mRFP chimera) were used and the fluorescent intensity at the red channel of tandem mRFP was about 3-fold that of EGFP. Thus, during FCCS analysis, we chose the cells having the roughly the same green and red fluorescent intensity ratios as the EGFP-mRFP chimera (the ratio of count rate for red/green: 2.6–3.4). The cross-correlation for the negative control measurement could be considered as the background, however, it could be attributable to the leakage of one fluorescence emission through another fluorescence detector as described previously [15]. The uniform ratio between red/green signals can correct the effect of the fluorescence leakage from another channel on the cross-correlation.

**Figure 3:**
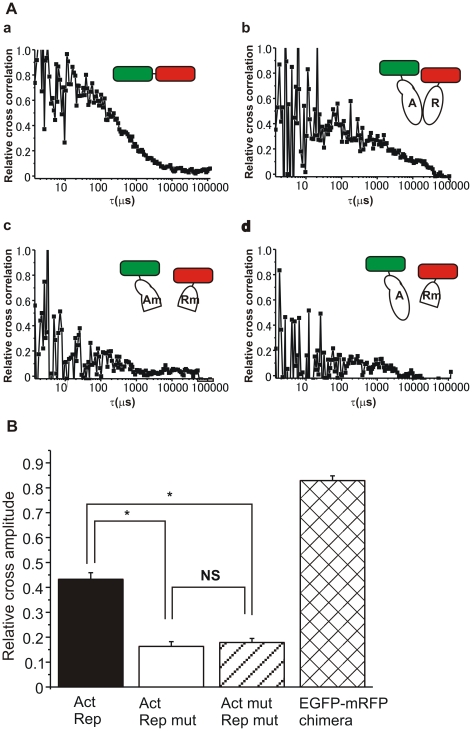
The quantitative evaluation of FCCS data for interaction between CREB1 isoforms. A, Relative cross-correlation [(*G*c(τ)−1)/(*G*r(0)−1)] calculated from FCCS measurement. Typical curves of cross-correlation were shown for EGFP- mRFP chimera (a), the EGFP-CREB1 activator isoform protein and the tandem mRFP-CREB1 repressor isoform protein (b), the EGFP-truncated CREB1 activator isoform protein and the tandem mRFP-truncated CREB1 repressor protein (c), and the EGFP-CREB1 activator protein and the tandem mRFP-truncated CREB1 repressor protein (d). B, Relative cross amplitudes [(*G*c(0)−1)/(*G*r(0)−1)] calculated from each FCCS measurement that corresponds to the fraction of the associated molecules (*N*_c_/*N*_g_). Here *N*_c_ is the average number of particles that have both green and red fluorescence in the excitation-detection volume, and *N*_g_ is that of the green fluorescent particles. Each relative cross amplitude: EGFP-Act and mRFP-Rep (black bar), EGFP-Act mut and mRFP-Rep mut (white bar), EGFP-Act and mRFP-Rep mut (hatched bar), EGFP-mRFP chimera proteins (cross-hatched bar).

We next performed the normalization of cross-correlation data among various samples. Following the previous report [16], the cross-correlation [*G*c(τ)−1] was normalized by [*G*r(0)−1] as the relative cross-correlation [*G*c(τ)−1]/[*G*r(0)−1] (Fig. 3A). As a result, we observed clear cross-correlation in the measurements of positive control experiments (Fig. 2A, 3Aa) and differently labeled CREB1 isoforms (EGFP-Act and mRFP-Rep, Fig. 2B, 3Ab), whereas, almost none of cross-correlation in the negative control experiments using truncated CREB1 isoform proteins (EGFP-Act mut and mRFP-Rep mut, and EGFP-Act and mRFP-Rep mut, Fig. 2C, 2D, 3Ac, 3Ad). The fluorescence auto-correlation functions of the green and red fluorescent molecules, *G*g(τ) and *G*r(τ) (τ: the time delay), and the fluorescence cross-correlation function, *G*c(τ), were calculated by equation (1) in Materials and Methods.

For the quantitative evaluation of cross-correlations, the cross-correlation amplitude [*G*c(0)−1] was normalized by [*G*r(0)−1] as the relative cross amplitude [*G*c(0)−1]/[*G*r(0)−1] (Fig. 3B). The relative cross amplitude of the positive control was 0.83±0.02 (EGFP-mRFP chimera proteins, n = 29), and that of CREB1 activator and repressor isoforms was 0.43±0.03 (EGFP-Act and mRFP-Rep, n = 20). The relative cross amplitudes of CREB1 activator and repressor isoforms (EGFP-Act and mRFP-Rep) were significantly higher than those of negative control samples [EGFP-Act and mRFP-Rep mut, 0.15±0.02, n = 10; EGFP-Act mut and mRFP-Rep mut, 0.18±0.02, n = 15; *P*<0.001 for each comparison]. The supplemental experiments also showed that the relative cross amplitudes of CREB1 heterodimer were almost the same as those of CREB1 homodimers (Fig. S1). More, the negative control experiments showed similar low values of relative cross amplitude (*P*>0.5), and the values were also similar to those of independent fluorescent molecules in a single cell reported previously [12], [16]. Thus, mRFP-Rep mut lacking the bZIP domain interacted to neither EGFP-Act nor EGFP-Act mut. In addition, interaction between EGFP-Act mut and mRFP-Rep was not also detected by FCCS analysis (data not shown). These results clearly showed that the high cross-correlation amplitude of EGFP-Act and mRFP-Rep is caused by heterodimer formation via specific interaction between the bZIP domains of CREB1 isoforms, but not to be the cause of the nonspecific protein interaction.

## Discussion

In this paper, we directly assessed the interaction between CREB1 activator and repressor isoforms in living cells using fluorescence cross-correlation spectroscopy (FCCS). In the nuclei of cells expressing EGFP- or mRFP-labeled intact CREB1 isoforms, strong interaction between green and red fluorescent proteins were observed as cross correlation (EGFP-Act and mRFP-Rep, Fig. 2B, 3Ab). The positive control experiments using EGFP-mRFP fusion protein supported that the high cross correlation ratio is the result of interaction between different fluorescent molecules (Fig. 2A, 3Aa). More, the negative control experiments using the truncated CREB1 proteins lacking the bZIP domains showed that the interaction is a consequence of dimer formation by the bZIP domains (Fig. 2C, 3Ac). Nor can the detected cross-correlation be attributed to the nonspecific interaction between CREB1 isoforms (Fig. 2D, 3Ad, Fig. S1). The results conclusively demonstrated that the high cross-correlation of FCCS analysis arose from the dimer formation between the bZIP domains of CREB1 isoform proteins (Fig. 3B).

The FCCS is an emerging technique that can physically and quantitatively evaluate molecular interaction [12], [13], [15]. Recently, FCCS has been applied to living cells to characterize molecular interactions directly in the intracellular environment [14], [17], [18]. For example, the transcription activator proteins Fos and Jun were examined for dimerization [15], for which *in vitro* studies had shown heterodimer formation during the course of their action. As Bacia and Schwille [14] mentioned, FCCS is suitable for detecting relatively strong interactions, such as “binding”, but not weak interactions. In line with the previous suggestion, our results by FCCS analysis clearly showed strong interaction between different CREB isoforms. We also performed FCCS analysis of identical CREB1 isoforms and observed that the relative cross amplitudes of homodimers were almost the same as those of heterodimer (Fig. S1). The result indicates the dimerization abilities of CREB1 isoforms are equal.

Another interesting finding is that the truncated CREB1 isoforms lacking a dimerization domain (CREB1-Act mut and CREB1-Rep mut) were observed slightly in cytoplasm, unlike the case with intact CREB1 proteins (CREB1-Act and CREB1-Rep) localized mainly in the nuclei (Fig. 1B). The truncated CREB1 proteins contained the nuclear transport signals as intact proteins (Fig. 1A), thus, the difference in the localization pattern indicates that the dimerization has some role in the localization or stabilization of CREB1 proteins in the nuclei. The previous studies suggested that CREB1 activator and repressor proteins interact with each other by making heterodimers, and that the interaction among CREB1 proteins is independent of the phosphorylation state as they are being activated for gene induction *in vitro* [9], and Wu et al. [19] reported that CREB dimerization is DNA-dependent. Taken together with these previous ideas, a possibility emerges that dimerization acts positively on the nuclear localization of CREB1 proteins to immobilize them in the nuclei, for instance, by binding them to DNA.

Additionally, because CREB1 proteins strongly interact to each other as shown in this study, the quantitative regulation of CREB1 activator and repressor will also be a critical component of CREB1-dependent gene regulation. However, FCCS analysis in this study clearly showed that the dimerization abilities were not different between heterodimer and homodimer (Fig. S1), indicating that CREB1 repressor does not actively absorb CREB1 activator to make heterodimers. Thus, the regulation of gene expression by CREB1 repressor can depend on the phosphorylation status of CREB1 activator, and depend on occupying CRE sites. In a previous study [11], we have quantified the mRNAs of all CREB1 isoforms in the snail brain, and confirmed that the CREB1 repressor, as well as the CREB1 activator, is constitutively expressed. The constitutive existence of the CREB1 activator and the repressor will offer an exquisite balance of the CREB1-mediated mechanism in gene expression. More, the CREB1 expression in the brain was upregulated after learning [11], indicating the molecular number of CREB is also regulated by extracellular stimuli. Correspondingly, cyclic AMP-responsive element modulator (CREM), another CREB family protein, makes various spliced isoforms, and there is a switch in CREM-gene expression during spermatogenesis resulting in the conversion of CREM from repressors to activators [20], [21]. Thus, spliced isoforms of CREB family proteins are also quantitatively regulated in the control of many biological events, and strong interaction between the isoforms will directly change the gene regulatory mechanism. One more interesting feature of CREB1 is the formation of dimerization with other family member proteins via the bZIP domain, such as CREB2 [2], [22], inducible cAMP early repressor (ICER) [23], [24], and CCAAT/enhancer binding protein [25], resulting in the wide functional ability of CREB1 proteins. The present study will also facilitate the future investigation of the molecular dynamics between CREB family proteins that accurately reflect those seen *in vivo*.

In conclusion, we clearly demonstrated the heterodimerization of the CREB1 activator and repressor by the cross-correlation between two fluorescent molecules observed in living cells, and this is the first report of direct observation of the interaction dynamics between CREB1 isoforms. The noninvasive analysis in living cells, as performed in the present study, will enable us to design future studies to investigate the function of CREB1 isoforms in a wide range cellular functions.

## Materials and Methods

### Plasmid construction and transfection procedure

The expression plasmids of CREB1 isoform proteins of *Lymnaea stagnalis* were constructed by PCR using the specific primer for each activator and repressor isoform. For the negative control experiment, plasmids that encode truncated CREB1 isoform proteins lacking dimerization regions were also constructed. Primers were designed according to the previous study [11], with the following sequences:

Act forward 5′- GATCTCGAGCTatgtcagcagggaatggt -3′,

Rep forward 5′- GATCTCGAGCTatggaagatgattcgaacag -3′,

reverse 5′- GCAGAATTCtcatgcatctttttgacagtataac -3′,

mut reverse 5′- CAGAATTCtcatttgacatactctttcttcttc -3′.

The primers “Act forward” and “reverse” were used for the subcloning of CREB1 activator (Act), “Rep forward” and “reverse” for CREB1 repressor (Rep), “Act forward” and “mut reverse” for truncated activator (Act mut), and “Rep forward” and “mut reverse” for truncated repressor (Rep mut), respectively. The PCR products of activator (Act) or its truncated protein (Act mut) for CREB1 isoforms was digested and ligated into the multiple cloning site of pEGFP-Cl (Clontech Laboratories, Mountain View, CA, USA), and the PCR products of repressor (Rep) or its truncated protein (Rep mut) was in the modified plasmid encoding tandem mRFP dimer. The CREB1 activator or repressor isoform protein was engineered in the C-terminal of EGFP or tandem mRFP.

Cell culture and transfection with plasmid DNA were performed as described previously [12]. HeLa cells grown on LAB-TEK chambered coverslips with eight wells (Nalge Nunc International, Naperville, IL, USA) were transfected using Effectene Transfection Reagent (Qiagen, Valencia, CA, USA). For the cross-correlation positive control experiment, the plasmid encoding EGFP and tandem mRFP fusion protein (EGFP-mRFP chimera) was used. The plasmids encoding the tandem mRFP dimer or EGFP-mRFP chimera were constructed in a previous work [12].

### FCCS measurements

FCCS was measured with a ConfoCor2 (Carl Zeiss, Jena, Germany) including two detectors of avalanche photodiodes (SPCM-200-PQ; EG&G, Vaudreuil, Quebec, Canada) equipped with an LSM510 inverted confocal laser scanning microscope (Carl Zeiss). EGFP was excited at the 488 nm laser line of a CW Ar^+^ laser, and mRFP was excited at the 543 nm laser line of a CW He-Ne laser through a water-immersion objective (C-Apochromat, 40×, 1.2NA; Carl Zeiss). Emission signals were split by a dichroic mirror (570 nm beam splitter) and detected at 505–530 nm for EGFP and 600–650 nm for mRFP. The confocal pinhole diameter was adjusted to 48 µm.

### Data analysis for FCCS

Auto-correlation curves for red and green channels, and a cross-correlation curve were obtained by using the algorithm described below in the software package for ConfoCor2 (Carl Zeiss) [15], [16], [26]. The fluorescence auto-correlation functions of green and red fluorescence, *G*g(τ) and *G*r(τ), and the fluorescence cross-correlation function, *G*c(τ), are calculated by
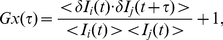
(1)where τ denotes the time delay; *I_i_* is the fluorescence intensity of the green fluorescence (*i* = g) or red fluorescence (*i* = r); and *G*g(τ), *G*r(τ), and *G*c(τ) denote the auto-correlation functions of green (*i = j = x* = g), red (*i = j = x* = r), and cross (*i* = r, *j* = g and *x* = c), respectively. Acquired *G*(τ) was fitted by a one-, two-, or three-component model as
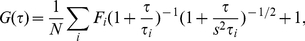
(2)where *F_i_* and τ*_i_* are the fraction and diffusion time of component *i*, respectively. *N* is the average number of fluorescent particles in the excitation-detection volume defined by radius w_0_ and length 2z_0_, and *s* is the structure parameter representing the ratio *s* = z_0_/w_0_. The average numbers of green fluorescent particles (*N*_g_), red fluorescent particles (*N*_r_), and particles that have both green and red fluorescence (*N_c_*) can be calculated by
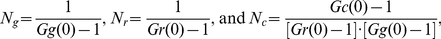
(3)respectively. When *N*_g_ and *N*_r_ are constant, *G*c(0) is directly proportional to *N*c. For evaluation of cross-correlation, cross-correlation amplitude [*G*c(τ)−1] is normalized by [*G*r(0)−1], i.e. the relative cross-correlation = [*G*c(τ)−1]/[*G*r(0)−1]. For the quantitative evaluation of cross-correlations among various samples, the cross-correlation amplitude [*G*c(0)−1] is normalized by [*G*r(0)−1], i.e., the relative cross-correlation amplitude = [*G*c(0)−1]/[*G*r(0)−1], which corresponds to the fraction of the associated molecules (*Nc*/*Ng*) [16].

### Statistics

The data are expressed as mean±SEM. Statistical significance was examined by a one-way ANOVA with Scheffe's post hoc test.

## Supporting Information

Figure S1 The quantitative evaluation of FCCS data for interaction between different or identical CREB1 isoforms. Relative cross amplitude [(*G*c(0)−1)/(*G*r(0)−1)] was calculated from each FCCS measurement and presented as the mean ± SE (n = 11–17). Each relative cross amplitude: EGFP-Act and mRFP-Rep (black bar), EGFP-Act and mRFP-Act (left hatched bar), EGFP-Rep and mRFP-Rep (right hatched bar), EGFP-Act mut and mRFP-Act (left open bar), and EGFP protein and mRFP-Act (right open bar). Black bar: CREB1 heterodimer. Hatched bar: CREB1 homodimer. Open bar: negative control. *, *P* <0.001 vs. negative controls.
(TIF)
